# Effectiveness of exercise interventions on mental health and health-related quality of life in women with polycystic ovary syndrome: a systematic review

**DOI:** 10.1186/s12889-021-12280-9

**Published:** 2021-12-20

**Authors:** Rhiannon K. Patten, Michaela C. Pascoe, Alba Moreno-Asso, Russell A. Boyle, Nigel K. Stepto, Alexandra G. Parker

**Affiliations:** 1 Institute for Health and Sport, Victoria University, Melbourne, Victoria Australia; 2 Peter MacCallum Cancer Centre, Victoria Melbourne, Australia; 3 Australian Institute for Musculoskeletal Science (AIMSS), Melbourne, Victoria Australia; 4 Centre for Youth Mental Health, University of Melbourne, Melbourne, Victoria Australia

**Keywords:** Exercise, physical activity, mental health, health-related quality of life, depression, anxiety

## Abstract

**Background:**

Polycystic ovary syndrome (PCOS) is a complex condition, impacting cardio-metabolic and reproductive health, mental health and health-related quality of life. The physical health benefits of exercise for women with PCOS are well-established and exercise is increasingly being recognised as efficacious for improving psychological wellbeing. The aim of this review was to summarise the evidence regarding the effectiveness of exercise interventions on mental health outcomes in women with PCOS.

**Methods:**

A systematic search of electronic databases was conducted in March of 2020. Trials that evaluated the effect of an exercise intervention on mental health or health-related quality of life outcomes in reproductive aged women with diagnosed PCOS were included. Methodological quality was assessed using the modified Downs and Black checklist. Primary outcomes included symptoms of depression and anxiety, and health-related quality of life.

**Results:**

Fifteen articles from 11 trials were identified and deemed eligible for inclusion. Exercise demonstrated positive improvements in health-related quality of life in all of the included studies. Half of included studies also reported significant improvements in depression and anxiety symptoms. There was large variation in methodological quality of included studies and in the interventions utilised.

**Conclusions:**

The available evidence indicates that exercise is effective for improving health-related quality of life and PCOS symptom distress. Exercise also shows some efficacy for improving symptoms and/or prevalence of depression and anxiety in women with PCOS. However, due to large heterogeneity of included studies, conclusions could not be made regarding the impact of exercise intervention characteristics. High-quality trials with well reported exercise intervention characteristics and outcomes are required in order to determine effective exercise protocols for women with PCOS and facilitate translation into practice.

**Supplementary Information:**

The online version contains supplementary material available at 10.1186/s12889-021-12280-9.

## Background

Polycystic Ovary Syndrome (PCOS) is a complex and common condition, affecting 8-13% of reproductive aged women [1] and carries a major disease burden across cardio-metabolic and reproductive health. PCOS is characterised by hyperandrogenism, ovulatory dysfunction and polycystic ovary morphology [2] and although not recognised in the diagnostic criteria, insulin resistance is considered a key aetiological feature, contributing to the severity of PCOS features [3]. PCOS is the leading cause of anovulatory infertility among reproductive-aged women [4] and has significant metabolic features including insulin resistance, obesity, and an increased risk of developing type 2 diabetes [5–8]. PCOS is also known to be related to diminished mental health, including increased symptoms of depression, anxiety and lower health-related quality of life, with these comorbidities occurring and having impact across the lifespan [9].

Many chronic illnesses have an impact on mental health and are associated with a reduction in quality of life and an increase in a range of psychological symptoms [9–11]. Given the clinical features of PCOS, it is perhaps not surprising that women with PCOS experience mental health problems and mood dysfunction to a greater degree than women without PCOS [12]. Compared to age and weight matched control women [13, 14], and those with other chronic conditions including diabetes and coronary heart disease [15], women with PCOS have poorer mental health and health-related quality of life with many reporting increased symptoms of anxiety and depression. Fears regarding infertility, body image concerns, low self-esteem and coping with the condition may all contribute to poorer mental health among these women [16]. In addition, the symptoms associated with PCOS often cause distress, leading to a reduced quality of life [17]. Symptom distress is often measured in women with PCOS using the polycystic ovary questionnaire (PCOSQ) which is a reliable instrument for measuring health-related quality of life in women with PCOS [17].

In a healthy population, exercise is an effective means of promoting, improving and managing mental health [18]. This is also the case for populations with chronic conditions [19] and in overweight women [20]. The specific interaction between exercise and mental health in PCOS has not been explored in depth, but the limited existing research indicates a positive effect of exercise for improving mental health and health-related quality of life in women with PCOS [21–24]. Women with PCOS who are more physically active report fewer symptoms of depression than sedentary women with PCOS [9], although active women with PCOS report higher symptoms of depression than active women without PCOS [9].

The current international evidence-based guidelines for the assessment and management of PCOS recommend 150 minutes per week of moderate intensity exercise or 75 minutes per week of vigorous intensity exercise in all women with PCOS, in order to improve general health and quality of life [1]. It is well documented that exercise elicits a number of health benefits including increased insulin sensitivity, increased cardiorespiratory fitness, improved menstrual cyclicity and improved mental health [21, 22, 25, 26]. Despite the positive effects of exercise, low compliance with these guidelines because of general barriers (time limitations, low enjoyment experienced with exercise) and PCOS-specific barriers (low confidence, physical limitations) to exercise [9, 21], means that many women with PCOS remain sedentary or insufficiently active (do not meet the minimum exercise recommendations) [27]. Enhancing engagement in exercise is vital to increase adherence to exercise recommendations and increase the potential health and mental health benefits of exercise [16]. This systematic review will synthesise the existing literature and aim to determine the effectiveness of exercise for improving symptoms of mental health and health-related quality of life in women with PCOS.

## Methods

### Protocol and registration

This systematic review was registered on the International Prospective Register of Systematic Reviews (CRD42019118657) and conducted in accordance with the Preferred Reporting Items for Systematic Review and Meta-Analyses (PRISMA) guidelines [28] and

### Information sources & search

An electronic database search was initially conducted in December 2018 and updated in October 2021 using Ovid Medline and EBSCOhost (PsycINFO, MEDLINE, SPORTDiscus, CINAHL), with no date or language restrictions. The search strategy included Medical Subject Heading (MeSH) terms and keywords relating to mental health, health-related quality of life, physical activity or exercise interventions and PCOS. An example of a search strategy is reported in Supplementary Table 1 and was adapted for each database. The search terms were broad in order to capture publications that may have included mental health or health-related quality of life as secondary outcomes. Reference lists of relevant articles were searched to identify additional eligible studies.

### Eligibility criteria

Included studies involved women of reproductive age (18-45 years of age) and with a diagnosis of PCOS using any established diagnostic criteria (e.g., Rotterdam criteria, National Institute for Health [NIH]). Randomised controlled trials (RCT), non-randomised controlled trials and uncontrolled trials were included. Exercise interventions of two weeks or greater were included in this review (Table 1). Exercise intensity was classified as moderate (55 to <70% HR_max_ or 40 to < 60% VO_2max_), vigorous (70 to <90% HR_max_ or 60 to <85% VO_2max_) or high intensity (≥90% HR_max_ or ≥85% VO_2max_) [29].

**Table 1:** Eligibility criteria for study inclusion

Participants	Intervention	*Comparison	Outcome	Study Design
Diagnosed with PCOS using any established definition Reproductive years, aged 18–45	Any intervention that included exercise of: Any type or intensity Duration >2 weeks	No exercise Alternative therapies (e.g. acupuncture, cognitive behavioural therapy) Medications	Depression symptoms Anxiety symptoms HRQoL (SF-36) PCOS symptom distress (PCOSQ)	RCT Non-RCT Cohort Case Control Parallel Clinical trial

PCOS – Polycystic Ovary Syndrome HRQoL – Health-Related Quality of Life, SF-36 – Short Form 36, PCOSQ – Polycystic Ovary Syndrome Questionnaire, RCT – Randomised Controlled Trial.

*Studies with no comparison were also included in this review.

### Outcomes

The outcome measures were health-related quality of life as assessed by either the polycystic ovary syndrome questionnaire (PCOSQ) or the short form 36 (SF-36) questionnaire and symptoms of depression and anxiety assessed by any validated questionnaire (Table 1). Trials that did not report at least one of these outcome measures were not included in this review.

### Study selection and data extraction

After duplicates were removed, two reviewers (R.P. and R.B.) independently screened each article by title and abstract. Following removal of irrelevant studies, full-text versions of the remaining publications were assessed for inclusion eligibility. Data relating to study design, participant and intervention characteristics, and outcome measures were extracted independently by reviewers using a pre-determined data extraction form. At each stage of the screening process, discrepancies were resolved by consensus or by a third reviewer (N.S.).. Due to poor reporting of intervention characteristics and outcome measures and large heterogeneity in the interventions, a quantitative synthesis was not feasible. Study results were therefore summarised as statistically significant within group changes (p<0.05).

### Risk of bias

The modified Downs and Black checklist for the assessment of methodological quality was used to evaluate the quality of included studies [30] (Supplementary Table 2). Questions regarding blinding of participants were removed as blinding is not possible in exercise intervention trials; however, blinding of outcome assessors was included. This checklist included 21 items with each item receiving a 0 or a 1 response and assesses reporting, internal and external validity or bias, and power. Higher scores indicated better methodological quality. Inter-reviewer discrepancies concerning the methodological quality of included studies were resolved by consensus.

## Results

**Fig. 1:**
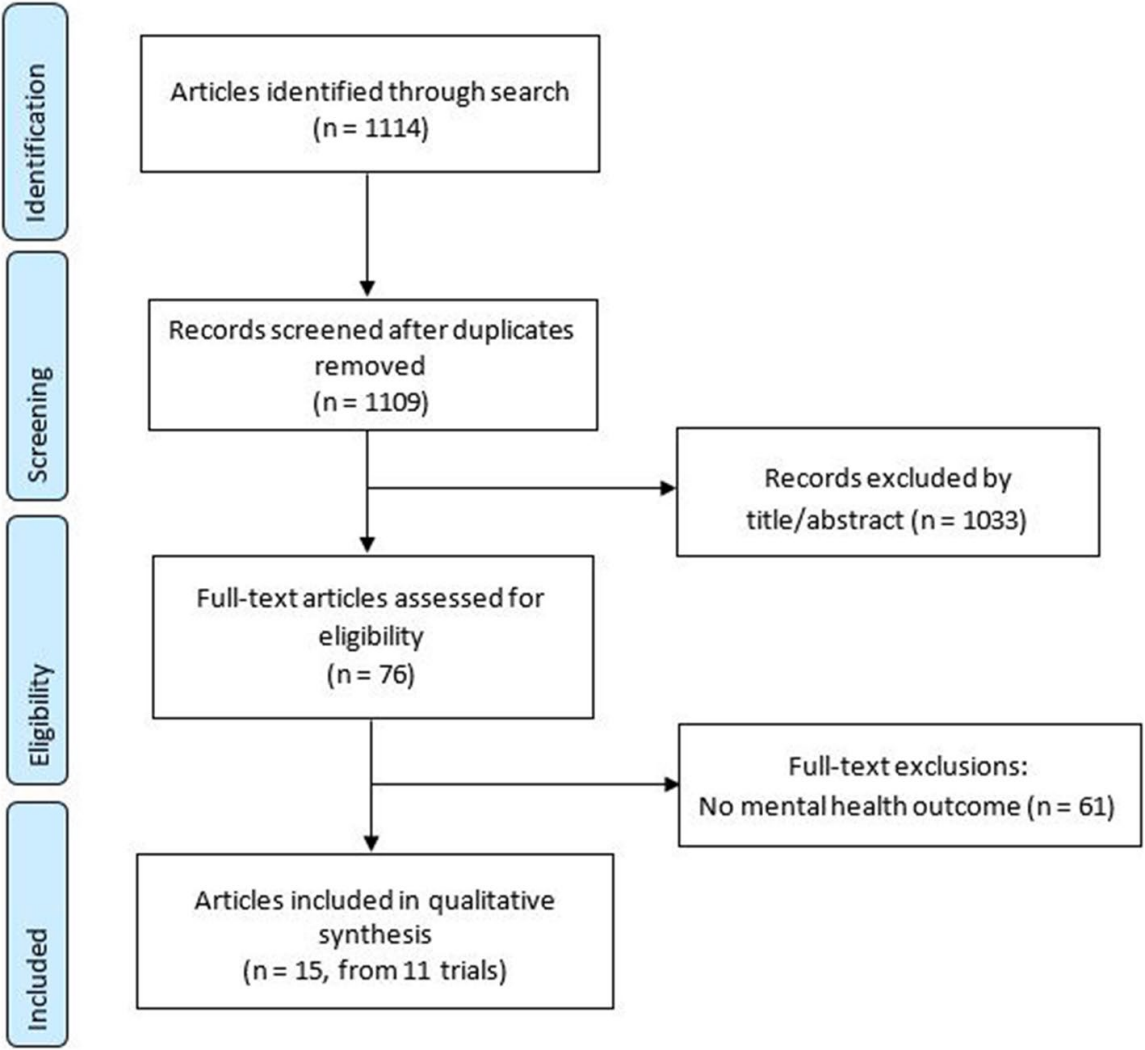
Preferred Reporting Items for Systematic Reviews and Meta-Analyses (PRISMA) study selection flow diagram

The database searches identified 1114 references. Six articles were removed due to duplication and 1033 articles were deemed irrelevant after title and abstract screening. Of the 76 papers that were deemed eligible for full-text screening, 61 were excluded due to having no relevant outcome measures (Fig. 1). The remaining 15 publications were deemed eligible for inclusion and were assessed for methodological quality, with results reported in Supplementary Table 2. These 15 publications were the result of 11 trials. In cases where multiple publications arose from one trial, data were grouped together. No additional studies were identified from the searches of reference lists of relevant studies. The characteristics of the included trials are presented in Table 2 and summarised below.

**Table 2:** Summary of studies identified for systematic review detailing participant and intervention characteristics, measures used and psychological outcomes.

Study	QA score^a^	Study Design	Exercise Intervention N (total N)	Participant Characteristics	Exercise Intervention(s) Characteristics	Comparison(s)	Measures	Mental health and health-related quality of life outcomes
Arentz et al. 2017 [31]	18	RCT	62 (122)	Age: 28.9 ± 5.6 years BMI: 35.2 ± 6.8 kg/m^2^ PCOS diagnostic criteria: Rotterdam	Type: Aerobic Frequency: N/R (90-150mins/week) Intensity: 60-90% HR_max_ Duration: 12 weeks Supervision: Partial	Herbal medicine + lifestyle intervention	PCOSQ DASS-21	^b^Significant improvements were seen for all domains of the PCOSQ and DASS-21 in the herbal medicine plus lifestyle group, significant improvements only for infertility (p=0.001), weight (p=0.01), menstrual problems (p=0.02) and emotions (p=0.04) in the lifestyle only group. No significant changes in DASS-21 scores in the lifestyle only group.
Cooney et al. 2018 [32]	13	Pilot RCT	8 (15)	Age: 32 (27-34) years BMI: 35 (31-40) kg/m^2^ PCOS diagnostic criteria: NIH	Type: Aerobic Frequency: N/R (50-175mins/week) Intensity: N/R Duration: 16 weeks Supervision: None	CBT (weekly 30min CBT sessions) + lifestyle modification	PCOSQ CES-D	Clinically but not statistically significant improvements in all domains of the PCOSQ (≥0.5 point increase) with the exception of menstrual problems in the overall group. Statistically significant improvement in depression scores (p=0.01) in the overall group, with no differences between groups (p=0.68).
Costa et al. 2018 [33]	16	RCT	14 (27)	Age: 27.6 ± 4.5 years BMI: 32 ± 4.2 kg/m^2^ PCOS diagnostic criteria: Rotterdam	Type: Aerobic Frequency: 3/week Intensity: 60-85% HR_max_ Duration: 16 weeks Supervision: Full	No intervention control group	SF-36	Significant improvements in physical functioning (p=0.004), general health (p=0.012) and mental health (p=0.042) domain scores compared to baseline.
De Frène et al. 2015 [34]	7	Single arm study	23	Age: 29 (5) years BMI: 33.7 (7.8) kg/m^2^ PCOS diagnostic criteria: Rotterdam	Type: Aerobic Frequency: N/R Intensity: N/R Duration: 24 weeks Supervision: None	None	PCOSQ	Significant positive effect on total PCOSQ score (p=<0.001) as well as emotions (p=<0.01), weight (p=<0.001), body hair (p=<0.05) and infertility (p=<0.001) domain scores.
Ladson et al. 2011 [35]	16	RCT	16 (26)	Age: 28.8 ± 4.6 years BMI: 38.3 ± 8 kg/m^2^ PCOS diagnostic criteria: NIH	Type: Aerobic Frequency: ≥2/week Intensity: N/R Duration: 26 weeks Supervision: Partial	Metformin + caloric restriction & exercise	PCOSQ	Significant improvements in emotions (p=0.008) and weight (p=0.002) domain scores.
Lara et al. 2015 [36] & Ramos et al. 2016 [37]	12 13	Case-control	43	Age: 27.9 ± 5.3 years BMI: 27.9 ± 5.5 kg/m^2^ PCOS diagnostic criteria: Rotterdam	Type: RT Frequency: N/R Intensity: 60-85% of 1RM Duration: 16 weeks Supervision: Full	Non-PCOS	HADS SF-36	Significant improvements in both anxiety (p=<0.01) and depression (p=<0.01) scores over time [36]. Significant improvements in SF-36 physical functioning domain (p=0.02) [37].
Legro et al. 2015 [24] & Dokras et al. 2016 [38]	18 12	RCT	49 (149)	Age: 28.6 ± 3.4 years BMI: 35.1 ± 4.6 kg/m^2^ PCOS diagnostic criteria: Rotterdam	Type: Aerobic Frequency: 5/week Intensity: N/R Duration: 16 weeks Supervision: None	OCP or combined OCP + lifestyle intervention	PCOSQ SF-36 PRIME-MD	Significant positive effect on weight (p=<0.0001), infertility (p=<0.0001), menstrual problems (p=0.004) PCOSQ domains [24]. Significant improvement in general health (p=<0.05) and vitality (p=<0.05) domains of the SF-36. Significant decrease in the prevalence of anxiety (15.9 to 4.7%; p=0.02). Non-significant changes in the prevalence of depression (22.7 to 15.9%; p=0.17) [38].
Ribeiro et al. 2019 [39] & Kogure et al. 2020 [40]	17 17	RCT	CAT = 28, IAT = 29 (87)	CAT = Age: 29.1 (5.3) years BMI: 28.4 (5.6) kg/m^2^ IAT = Age: 29.0 (4.3) years BMI: 28.7 (4.8) kg/m^2^ PCOS diagnostic criteria: Rotterdam	Type: Aerobic Frequency: 3/week Intensity: CAT – 65-80% HR_max_ IAT – 70-90% HR_max_ Duration: 16 weeks Supervision: Full	No intervention control group	SF-36 HADS	CAT – Significant improvements in physical functioning (p=0.022), role physical (p=<0.001), general health (p=<0.001), vitality (p=<0.001), social functioning (p=<0.001), role emotional (p=<0.001) and mental health (p=<0.001) domains of the SF-36. IAT – Significant improvements in physical functioning (p=<0.001), role physical (p=0.027), general health (p=<0.001), vitality (p=0.001), social functioning (p=<0.001), role emotional (p=0.011) and mental health (p=<0.001) domains of the SF-36 (36). Significant improvements in anxiety and depression scores (p=<0.05) in both the CAT and IAT groups [40].
Stener-Victorin et al. 2013 [41]	13	RCT	29 (44)	Age: 29.9 ± 4.4 years BMI: 28.1 ± 7.4 kg/m^2^ Diagnostic criteria: Rotterdam	Type: Aerobic Frequency: ≥3/week Intensity: N/R Duration: 16 weeks Supervision: None	No intervention control group & acupuncture group	MADRS-S BSA-S PCOSQ SF-36	No significant improvements in anxiety or depression. Significant improvements in PCOSQ domains for infertility (p=<0.05) and emotions (p=<0.001) and the role physical (p=<0.001) domain of the SF-36.
Thomson et al. 2010 [22] & Thomson et al. 2016 [42]	12 12	RCT	Aerobic only = 15 Aerobic + RT = 20 (49)	Age: 29.3 ± 6.8 years BMI: 36.1 ± 4.8 kg/m^2^ Diagnostic criteria: Rotterdam	Type: Aerobic only or combined aerobic & RT Frequency: 5/week Intensity: Aerobic = 60-80% HR_max_, RT = 50-75% of 1RM Duration: 20 weeks Supervision: Partial	^c^Diet only (energy restricted, high protein diet)	CES-D PCOSQ	Significant improvement in depression scores in all groups (p=≤0.001) with no effect of treatment (p=0.86). Significant improvements in PCOSQ domain scores for emotions (p=≤0.001), weight (p=≤0.001), menstrual problems (p=≤0.001), and infertility (p=≤0.001) for all groups.
Vizza et al. 2016 [23]	19	Pilot RCT	7 (13)	Age: 26.7 ± 7 years BMI: 41.3 ± 12.5 kg/m^2^ Diagnostic criteria: Rotterdam	Type: RT Frequency: 2/week Intensity: N/R Duration: 12 weeks Supervision: Full	No intervention control group	PCOSQ SF-36 DASS-21	Significant improvements in the RT group compared to the control group for emotions (p=0.003), weight (p=0.04) and infertility (p=0.03) PCOSQ domains. Significant improvements in the RT group compared to the control group for physical functioning (p=0.02), vitality (p=0.02), social functioning (p=0.002), role emotional (p=0.009) and mental health (p=0.009) SF-36 domains. Significant improvements in the RT group compared to the control group for depression (p=0.01) and anxiety (p=0.03).

Data presented as mean ± SD or median (IQR).

1RM – One Repetition Maximum, BMI – Body Mass Index, BSA-S – Brief Scale for Anxiety, CAT – Continuous Aerobic Training, CBT – Cognitive Behavioural Therapy, CES-D – Centre for Epidemiological Studies Depression scale, DASS-21 – Depression, Anxiety and Stress Scale 21, HADS – Hospital Anxiety and Depression Scale, HR_max_ – Maximum Heart Rate, IAT – Intermittent Aerobic Training, MADRS-S – Montgomery Åsberg Depression Rating Scale, N/R – Not Reported, OCP – Oral Contraceptive Pill, PCOSQ – Polycystic Ovary Syndrome Questionnaire, PRIME-MD - Primary Care Evaluation of Mental Disorders, QA – Quality Appraisal, RCT – Randomised Controlled Trial, RT – Resistance Training, SF-36 – Short Form 36.

^a^Methodological quality score from the Downs and Black checklist. Possible range of scores 0-21.

^b^Data provided by author.

^c^All groups received the diet intervention.

### Study design and participants characteristics

Of the 11 included trials, nine were RCTs [22–24, 31–33, 35, 39, 41], one was a single arm study [34] and one was a case-control study [36]. Sample sizes ranged from 13 to 149 participants. The mean age of participants ranged from 26 to 33 years of age and the mean body mass index (BMI) ranged from 27.9 to 41.3kg/m^2^. Participants from nine trials were diagnosed with PCOS according to the Rotterdam criteria [22–24, 31, 33, 34, 36, 39, 41] and three trials according to the NIH criteria [32, 35]. Of the included trials, nine recruited only overweight and obese women with PCOS and all studies excluded women with chronic co-morbidities, such as diabetes and cardiovascular disease. One study included only women with a positive screen for depression symptoms [32]. Three studies excluded women who were taking medication treatment for depression [22, 31, 43], five studies did not exclude women who were taking anti-depressant medication [23, 24, 36, 39, 41] and two studies did not report on use of medication for mental health status [34, 35], as shown in Table 2.

### Assessment of study quality

Scores from the modified Downs and Black checklist varied greatly with scores ranging from 7 to 19 on a 21-point scale, with a lower score indicating a poorer methodological quality. The most common issues were poor reporting of aspects of the intervention or the characteristics of participants lost to follow-up. The full assessment of methodological quality is presented in Supplementary Table 3.

### Intervention characteristics

Aerobic exercise alone was delivered in eight out of the 11 trials [24, 31–35, 39, 41]. One study included an aerobic exercise group, a resistance training group and a combined aerobic and a resistance training group [22] and the final two studies delivered resistance training only [23, 36]. Of the included studies, only four of the interventions had full supervision by an exercise specialist [23, 33, 36, 39], three had partial supervision [22, 31, 35] and four had no supervision [24, 32, 34, 41]. The duration of the interventions varied from 12 to 26 weeks, with the number of sessions ranging from two to five per week. Exercise intensity was only adequately reported in five of the 11 included studies, all of which reported using moderate-vigorous intensity exercise. Adherence, classified as the percent of sessions attended in regards to the expected attendance, was reported in only three of the included studies. Two of these reported an average adherence of 81% [33] and 95% [23] for supervised sessions, with one of these trials also reporting an average of 51% for unsupervised session adherence [23]. The third study reported that on average, only 38% of participants in the lifestyle only group reported meeting their weekly exercise goal [32]. No adverse events were reported as a result of any of the exercise interventions.

### Outcomes

Six of the included trials had a primary outcome of mental health and/or health-related quality of life. The remaining five trials included mental health and/or health-related quality of life as secondary outcome.

### PCOS symptom distress

Of the studies included, eight used the PCOSQ to assess PCOS related distress, seven of which reported statistically significant improvements in a minimum of two domains. The PCOSQ is a validated questionnaire which assessed the multidimensional concept of health-related quality of life, encompassing five domains; emotions, hair growth, body weight, infertility and menstrual problems. A higher overall score indicates better function, and a change of 0.5 is considered clinically relevant [44]. Six studies reported statistically significant improvements in emotions [22, 23, 31, 34, 35, 41] weight [22–24, 31, 34, 35] and infertility domains [22–24, 31, 34, 41], three reported improvements in the menstrual problems domain [22, 24, 31] and one reported improvements in the body hair domain [34]. The eighth study did not report a statistically significant impact of exercise on PCOS related distress, but did report minimally important clinically significant improvements (≥0.5 point increase), in all domains for the cognitive behavioural therapy plus lifestyle group, and clinically significant increases in the weight and infertility domains for the lifestyle only group [32]. In total, the weight domain had the most clinically significant improvements with six studies reporting improvements ranging from 0.7 to 1.75 point increase in scores. Clinically significant improvements were also commonly reported in the emotions domain, with four studies reporting increases in scores of between 0.5 and 0.8 points. A large proportion of interventions that utilised a range of exercise intensities, doses, types and durations, resulted in significant improvements in multiple domains of the PCOSQ, therefore suggesting that regardless of these factors, exercise can improve health-related quality of life in regards to PCOS symptom distress.

### Health-related quality of life

Six studies used SF-36 to determine the health-related quality of life of participants. The SF-36 measures eight dimensions of health; physical functioning, role limitations due to physical problems (role physical) , bodily pain, general health perception, vitality, social functioning, role limitations due to emotional problems (role emotional) and mental health [45]. Four studies reported statistically significant increases in the physical functioning domain [23, 37, 39, 43]. Three studies reported significant increases in general health [38, 39, 43], vitality [23, 38, 39] and mental health [23, 39, 43]. Two studies reported statistically significant improvements in role physical [39, 41], social functioning [23, 39] and role emotional domains [23, 39]. No studies reported statistically significant improvements in the bodily pain domain. Clinically meaningful improvements were most commonly reported for the role physical domain, with 4 studies showing clinically meaningful changes after an exercise intervention [37, 39, 41, 43], reporting increases in scores of 6.3 to 39.3. In regards to the SF-36, a change of score of 5 points or greater is considered clinically meaningful [46]. Improvements were also commonly reported for emotional and mental health domains with increases in scores of between 5.0 to 40.6, and 11.7 to 15.4 respectively. There were three studies that observed large improvements in multiple domains of the SF-36. Two of these studies used an aerobic exercise intervention of moderate to vigorous intensity, delivered three times per week for 16 weeks [39, 43], while the third study conducted a 12 week progressive resistance training program delivered twice per week [23]. In summary, many studies reported improvements in domains of the SF-36 as a result of various exercise interventions, however it appears that there were no common denominators in regards to exercise characteristics.

### Depression

Eight studies measured the effect of an exercise intervention on depression symptoms, with five reporting significant reductions in depression scores. Two studies that reported improvements delivered an aerobic exercise intervention [32, 40], two delivered resistance training [23, 36] and the final study compared three interventions (diet only, diet and aerobic exercise and diet and combined exercise) [22], all of which resulted in improved depression scores. There were no obvious common denominators in regards to exercise characteristics between studies that did, and those that did not report improvements in symptoms of depression. One study that used the Depression Anxiety and Stress Scales (DASS-21) questionnaire reported significant changes in depression symptoms at post-intervention, in comparison to a no-intervention control group, after 12 weeks of supervised resistance training [23]. One study, reported significant decreases in depression symptoms following a combined diet and aerobic intervention, with average scores on the CES-D decreasing from 18.6 at baseline to 14.0 post-intervention [22]. A second study that used the CES-D included only women classified as possibly depressed (score of ≥16) at baseline, and reported large decreases in symptoms with average scores decreasing from 24 to 18 [32]. Lastly, both studies that used the HADS questionnaire reported significant improvements in depression scores from baseline, one of which utilised a resistance training intervention [36] and the second study utilised two different aerobic training programs, both of which reported significant improvements [39].

### Anxiety

Six studies also examined symptoms of anxiety, three of which reported statistically significant within group reductions in symptoms after an exercise intervention [36, 38, 40]. A third study reported significant reductions in anxiety symptoms compared to a control group with scores reducing from 10.3 at baseline to 7.4 post-intervention [23]. Two studies reported the prevalence of anxiety at baseline and following an exercise intervention. One of these studies reported that 44.1% of participants had anxiety (according to the measures used) at baseline, which decreased to 23.2% after a 16 week resistance training intervention [36]. The second study reported a drop from 15.9 to 4.7% of women considered to have clinical anxiety after a 16 week aerobic exercise intervention [38].

### Summary

In summary, exercise interventions reduced symptoms of depression and anxiety in half of the reviewed studies. Due to the large variety of training interventions, conclusions cannot be made regarding the impact of a specific type or intensity of an exercise intervention, compared to another.

## Discussion

The current systemic review aimed to determine the effectiveness of exercise on mental health and health-related quality of life outcomes in women with PCOS. Exercise interventions appear to have positive effects on health-related quality of life and associated PCOS symptom distress as assessed by validated measures. The findings for mental health outcomes were less consistent, with a combination of positive and null findings regarding improvements in symptoms of anxiety and depression, although half of the included studies reported improvements in symptoms of anxiety and depression after an exercise intervention. The most common exercise program included various types of aerobic exercise of varying intensities, ranging from moderate to high intensity. Others included some form of resistance training program or a combination of resistance and aerobic exercise. Intervention duration and the inclusion of supervised exercise also varied among interventions. There did not appear to be any common exercise characteristics that could explain differences in symptoms of depression and anxiety, PCOS associated distress, or improvements in health-related quality of life outcomes.

This systematic review expands on an existing review of seven trials that found exercise to be beneficial for improving health-related quality of life, depression and anxiety in women with PCOS [21]. Future research is required however to provide further evidence of these benefits. Observed improvements resulted from various types of exercise, exercise intensities and concurrent therapies, making it difficult to determine the components of the intervention that contribute to improved outcomes. Studies included in this review were largely heterogeneous with varying interventions, concurrent therapies, sample sizes, study designs, comparator groups and methodological quality making the independent effect of any particular type of exercise intervention or characteristics difficult to assess. These variations prevented a meta-analysis from being conducted and limited the ability to form conclusions about the effectiveness of exercise on mental health and health-related quality of life in women with PCOS. In addition, poor reporting of exercise characteristics and the large variety of intensities, duration and frequency, limited the capacity to formulate more specific exercise recommendations for promoting mental health and health-related quality of life in women with PCOS, which limits the translation of these research findings into clinical practice.

The current international evidence based guidelines for the assessment and management of PCOS states that psychological factors, including anxiety and depression, should be screened, assessed and managed [1]. It is important to ensure positive well-being to increase quality of life but also to assist in promoting engagement and adherence to lifestyle interventions. Adherence to exercise interventions has been reported to be low in clinical settings among women with PCOS [35, 47, 48], it is crucial that future studies report measures of adherence to determine interventions that are more effective in maintaining the interest and enjoyment of participants. Very few of the studies reviewed in the current research reported on adherence and compliance to the exercise intervention. In the studies that did report on adherence, supervised sessions had a much greater attendance rate and could ultimately contribute to larger improvements in physical and mental health. Future studies should consider commencing with supervision of all exercise sessions, in order to address initial exercise engagement, followed by a tapering of supervision to include planned, unsupervised exercise, while concurrently promoting and encouraging self-sustainability, to promote long-term maintenance of exercise, following the completion of the intervention.

Given that previous research shows that time limitation is reported as the biggest barrier to exercise participation both in a general population and among women with PCOS [9], the alternative of a vigorous intensity or high intensity interval training may provide a solution to this barrier. Some, but not all, research suggests that individuals may experience greater enjoyment when partaking in high intensity exercise compared to continuous moderate intensity exercise [49–51]. Significant improvements following high intensity interval training have been reported in systematic reviews/meta-analyses for anxiety, depression [49, 52] and quality of life [25, 53] outcomes, however, these benefits have only been reported in patients with chronic conditions other than PCOS. In women with PCOS, there is limited evidence to suggest that high intensity exercise can result in greater health improvements compared to moderate intensity exercise [25]. However the potential effects of high intensity exercise, in particular, on mental health and health-related quality of life outcomes has yet to be thoroughly investigated. Thus, more studies examining the effects of high intensity exercise on mental health outcomes in women with PCOS are needed.

Half of the studies included in this review reported significant reductions in anxiety and depression symptoms after an exercise intervention, especially when considering that the reviewed interventions were primarily aimed at improving the physical health, rather than the mental health, of participants. Designing future interventions with a mental health informed rationale for the exercise intervention may improve engagement and therefore result in greater mental health benefits. For example, multi-component interventions that also include additional therapies such as cognitive behavioural therapy could be considered useful and may aid to increase adherence, retention, engagement as well as the maintenance of a healthy lifestyle to improve all health outcomes in women with PCOS [1]. Although this review was focused on the effects of exercise only, one of the included studies examined a multi-component intervention that included cognitive behavioural therapy, and reported clinically significant improvements in all domains of the PCOSQ and depression symptom scores [32]. Therefore, further examination of multi-component interventions could provide useful information for improving mental health for women with PCOS.

In addition, many of the included trials excluded women who were taking medications for the treatment of clinical anxiety and depression. Given the high prevalence of these two mental health conditions in women with PCOS, excluding these women does not adequately represent the population, and therefore, perhaps the true benefits of exercise for these women. We could benefit greatly from future research that examines the effect of exercise in women with PCOS, who display elevated baseline levels of anxiety and depression. Such research would provide greater insight regarding the efficacy of exercise for improving mental health and health-related quality of life in women with PCOS and would increase generalisability and applicability to real-world clinical practice.

### Strengths and Limitations

This review builds on existing knowledge and provides preliminary data to support the inclusion of exercise to manage and improve mental health and health-related quality of life outcomes in women with PCOS. A strength of this review is that it follows the PRISMA guidelines, including double screening of articles, data extraction and quality appraisal of each publication. This review was limited by large variations and poor reporting of exercise characteristics in the included primary studies. This hindered us from being able to conduct a quantitative synthesis of results and limited our ability to form strong conclusions about the effectiveness of exercise, and particular exercise characteristics, on mental health and health-related quality of life in women with PCOS. Future studies should endeavour to adequately report all intervention characteristics, including frequency, intensity, type, format and session duration of exercise interventions and as well as reporting both adherence and compliance to the exercise intervention inform future research.

## Conclusions

This review found that exercise results in both clinically meaningful and statistically significant improvements in health-related quality of life in women with PCOS. Exercise also appears to have some benefit for improving symptoms of common mental health concerns with half of studies reporting significant improvements in symptoms of depression and anxiety. However, the heterogeneity of included studies, including methodological quality, and the poor reporting of the characteristics of exercise interventions delivered, limited the ability to make conclusions regarding the effectiveness of specific types of exercise. This also limited the ability to conclude the impact of specific exercise characteristics including intensity, frequency and type of exercise for improving mental health in women with PCOS. It is vital to employ strategies that can both reduce symptoms of anxiety and depression and increase adherence to interventions. Therefore, multi-component interventions that integrate psychological treatment with exercise and address the complex physical and mental health concerns of women with PCOS have the potential for improving mental health outcomes. Future studies should aim to address barriers to exercise participation and determine which intervention characteristics are associated with increased engagement and maintenance of exercise for the promotion of mental health in women with PCOS.

## Supplementary Information

**Additional file 1.**

**Additional file 2.**

**Additional file 3.**

## Data Availability

Data sharing is not applicable to this article as no datasets were generated or analysed during the current study.

## List of abbreviations

1RM: One Repetition Maximum
BMI: Body Mass Index
BSA-S: Brief Scale for Anxiety
CAT: Continuous Aerobic Training
CBT: Cognitive Behavioural Therapy
CES-D: Centre for Epidemiological Studies Depression scale
DASS-21: Depression, Anxiety, Stress Scale-21
HADS: Hospital Anxiety and Depression Scale
HR_max_: Maximum Heart Rate
IAT: Intermittent Aerobic Training
MADRS-S: Montgomery Åsberg Depression Rating Scale
OCP: Oral Contraceptive Pill
PCOS: Polycystic Ovary Syndrome
PCOSQ: Polycystic Ovary Syndrome Questionnaire
PRIME-MD: Primary Care Evaluation of Mental Disorders
QA: Quality Appraisal
RCT: Randomised Controlled Trial
RT: Resistance Training
SF-36: Short Form-36
