# Supplementary material for: Effectiveness of exercise interventions on mental health and health-related quality of life in women with polycystic ovary syndrome: a systematic review
Source: BMC Public Health. 2021 Dec 20;21:2310. doi: 10.1186/s12889-021-12280-9 (PMC8690971; doi:10.1186/s12889-021-12280-9)
Supplement: Supplementary file 1 — Additional file 1. [file 12889_2021_12280_MOESM1_ESM.docx]

**Article title:** ‘Effectiveness of exercise interventions on mental health and health-related quality of life in women with polycystic ovary syndrome: a systematic review’

**Corresponding author:** Rhiannon Patten, [Rhiannon.patten@live.vu.edu.au](mailto:Rhiannon.patten@live.vu.edu.au).

**Supplementary Table 1** Search terms to identify exercise interventions in PCOS

| **PCOS** | **Exercise** | **Limits** |
| --- | --- | --- |
| Polycystic ovary syndrome  or  Polycystic ovar*  or  PCOS  or  Stein Leventhal | AND  Exercise  or  Physical  or  Sport  or  Strength  or  Resistance  or  Lifestyle | NOT  Insulin resistance |
